# Assistive technology-based solutions in learning mathematics for visually-impaired people: exploring issues, challenges and opportunities

**DOI:** 10.1007/s11042-023-17409-z

**Published:** 2023-10-25

**Authors:** Muhammad Shoaib, Donal Fitzpatrick, Ian Pitt

**Affiliations:** 1 School of Computer Science and Information Technology, University College Cork, Cork, Ireland; 2 Centre for Excellence in Universal Design, National Disability Authority, Dublin, Ireland

**Keywords:** Assistive Technology, Visually Impaired, Learning, Mathematics, Education, Accessibility

## Abstract

In the absence of vision, visually impaired and blind people rely upon the tactile sense and hearing to obtain information about their surrounding environment. These senses cannot fully compensate for the absence of vision, so visually impaired and blind people experience difficulty with many tasks, including learning. This is particularly true of mathematical learning. Nowadays, technology provides many effective and affordable solutions to help visually impaired and blind people acquire mathematical skills. This paper is based upon a systematic review of technology-based mathematical learning solutions for visually impaired people and discusses the findings and objectives for technological improvements. It analyses the issues, challenges and limitations of existing techniques. We note that audio feedback, tactile displays, a supportive academic environment, digital textbooks and other forms of accessible math applications improve the quality of learning mathematics in visually impaired and blind people. Based on these findings, it is suggested that smartphone-based solutions could be more convenient and affordable than desktop/laptop-based solutions as a means to enhance mathematical learning. Additionally, future research directions are discussed, which may assist researchers to propose further solutions that will improve the quality of life for visually impaired and blind people.

## Introduction

People may have various degrees of sight loss, ranging from low vision (usually defined as having a level of visual acuity in the better eye which falls below a certain standard despite correction) through to complete or nearly complete absence of vision. Blindness affects a significant minority of the human population. According to a report (WHO 2019), 2.2 billion people are estimated to be visually impaired worldwide and one billion of these have a vision impairment that is yet to be addressed [1].

Interaction with computers and smartphones is predominantly based on vision, limiting or precluding access for those who are visually impaired or blind. Similarly, in educational institutes, teaching methodologies are heavily dependent upon visual information, i.e., instructor gestures, slide shows, writing and sketching on a board. Visually impaired and blind people can use technology to perform daily routine tasks such as communication with their friends, accessing online information, and e-learning.

Mathematics plays an important role in education, from the primary level through to higher education. Mathematics requires an understanding of a number of concepts, including addition, subtraction, multiplication, division, expressions, formulas, logic, graphs, algebra and algorithms. Ideally, visually impaired students should learn these concepts in the early stages of their education, at the same time as their fully-sighted peers. However, mathematics has rich visual content and information which may present problems for visually impaired students [2]. During mathematical learning, visually impaired students may have difficulty recognizing symbols, identifying equations, understanding graphs, and performing arithmetic operations. Emerson et al. [3] noted that materials used in mathematics education may include visual images supporting related content, making it inaccessible for visually impaired students. Diagrams, figures and graphs are used to express the relationship between data in a way which is helpful for fully-sighted students but may present barriers for visually impaired students [4]. Mathematics students should be able to use the information presented in graphs, etc., and apply it in order to solve problems, but this may not be possible for visually impaired students [5, 6].

Computer-based learning has a positive impact on enhancing mathematical skills[7]. Continual enrichment in digital technologies increases the use of technological tools in mathematics learning [8–10]. Technology provides an opportunity for visually impaired students to access mathematical information using audio-based interfaces, tactile devices and Braille [11–17]. These Assistive Technology (AT) based learning tools can make educational platforms more interactive, engaging and easy to use for visually impaired and blind people, and can also facilitate collaboration. Web-based applications are also very useful because the material—provided it is correctly formatted—can be accessed by everyone regardless of their disability. Smartphone-based solutions are also of interest because, although smartphones may be less accessible and adaptable than other devices, they are also generally less expensive, have a wide range of features, and are widely used throughout the world, including by visually impaired and blind people [18]. These solutions have important features which are very useful for visually impaired and blind people, i.e., Text-to-Speech, Speak Selection, Voice Over/TalkBack, adjustable contrast, zoom and voice command along with several non-speech audio feedback options [19].

The previous surveys provide an overview of the currently available software tools for accessing mathematical documents and content. They also discussed the advantages and disadvantages of several key technologies used to interact with mathematics documents. Additionally, they review the importance of the most common formats and languages behind these tools. In this article, we are dealing with assistive technology-based solutions for learning mathematics for visually-impaired people and exploring the issues, challenges and opportunities in a broader scope. We have reviewed previous studies that explored experimental and commercial applications, running on various platforms (laptop/desktop, smartphones and the web) to illustrate how visually impaired and blind students can learn mathematics by using them. We have considered both general solutions and those designed to support specific tasks in order to obtain information on the accessibility of mathematics learning tools. We have investigated AT-based tools, techniques, algorithms, systems and projects, and provided a comparison of their major functions, the nature of the feedback they provide, input and output mechanisms, and the mathematical operations which they are designed to support. We have also considered the challenges that visually impaired individuals faced when learning mathematics using commercial and experimental smartphone applications and discussed how we can address these challenges in novel ways. In addition, we have extracted the features of these math learning applications and proposed a categorization of these solutions that we hope will prove useful to researchers and developers seeking to develop effective solutions for visually impaired and blind students. Based on the findings, recommendations are made for researchers who are working to enhance learning among visually impaired and blind people. Finally, this article discusses the need for further research to improve the development of mathematical software tools that are designed specifically for visually impaired and blind individuals, taking into account their unique needs and challenges.

The paper is organized as follows: Section 2 describes the methodology used for the review; Section 3 reviews relevant theoretical work, Section 4 provides an overview of experimental solutions proposed to assist blind and visually-impaired people in teaching or learning mathematics, both general solutions and those designed to support specific tasks; Section 5 discusses commercial and research-based applications available for learning mathematics; Section 6 presents a discussion of the findings; Section 7 concludes the current study and Section 8 contains recommendations for future research.

## Research Methodology

In our systematic literature review, we have followed the guidelines proposed by Kitchenham and Charters [20]. In this section, we have defined the research questions, search strategies, study selection criteria and classification of the research articles.

### Research questions

To assess the significance of technology on the development of mathematical abilities among visually impaired and blind students, we have addressed the following research questions:

- RQ1. What is the impact of technology on the development of mathematical abilities among visually impaired and blind students?
- RQ2. What are the challenges/issues facing visually impaired and blind students using commercial and researcher-based applications?
- RQ3. Can the development of smartphone-based applications be useful in helping visually impaired and blind people to learn mathematics?

### Search strategy

This study involved a systematic search of articles and studies that were published after January 1990. The targeted population of the study is visually impaired and blind people.

1. Identifying Search Terms
  For this study, we extracted major terms from the research questions and also explored the synonyms of these terms. We searched relevant research articles and verified the keywords. Additionally, we used Boolean operations, i.e., ‘OR’ for conjunction, ‘AND’ for concatenation.
2. Search Strings
  [(“Technology” OR “Assistive Technology” OR “Digital Tools”) AND (“mathematical ability” OR “math skill” OR “Learning mathematics”) AND (“visually impaired” OR “blind” OR “low vision”), AND (“Commercial Applications” OR “researcher-based application” OR “assistive technology”) AND (“visually impaired” OR “blind” OR “low vision”) AND (“challenges” OR “issues” OR “barriers” OR “obstacles”) AND (“mathematics” OR “math learning”), (“Smartphone-based application” OR “mobile application” OR “mobile learning”) AND (“visually impaired” OR “blind” OR “low vision”) AND (“mathematics” OR “math learning”) AND (“assistive technology” OR “accessibility”), (“Blind” OR “Visually Impaired” OR “Low Vision” OR “unsighted” OR “partially sighted”) AND (“Information Technology” OR “Innovative Technology” OR “Computer-Based system” OR “Virtual Reality” OR “Assistive Technology” OR “Smartphones” OR “games”) AND (“Learning mathematics” OR “Math skills” OR “Useful mathematics learning applications” OR “Helpful math learning applications” OR “Development of mathematical abilities”)].
3. Trial Search
  We conducted a trial search using the above-mentioned search strings on ResearchGate, Citeseer, Springer Link, Google Scholar, IEEE Xplore, ACM, Science Direct and Scopus digital libraries. Table 1 lists the number of results found on various electronic data sources.

**Table 1:** Trail search results and electronic data sources name

S.No	Digital library	Data sources Link	Search	Conduction Date
1	Researchgate	researchgate.net	60	01 May 2023
2	Citeseer	citeseer.ist.psu.edu	50	24 April 2023
3	SpringerLink	springerlink.com	421	05 May 2023
4	Google Scholar	scholar.google.com.pk	270	07 May 2023
5	IEEE Xplore	ieeexplore.ieee.org	220	05 May 2023
6	ACM	acm.org	30	09 May 2023
7	ScienceDirect	sciencedirect.com	30	15 April 2023
8	Scopus	scopus.com	240	01 April 2022

^a^https://play.google.com/store/apps/details?id=com.mathmelodies&hl=en&gl=US

^a^https://apps.apple.com/us/app/math-robot/id704570512

^a^https://play.google.com/store/apps/details?id=com.mediakube.touchmath.tcpatternspro

^a^https://apps.apple.com/us/app/uabacus/id688547692

^a^https://apps.apple.com/us/app/slapstack-math/id1209184917

^a^https://apps.apple.com/us/app/draw2measure-protractor/id1097557700

^a^https://apps.apple.com/us/app/practice2master-fractions/id1257764758

### Study Selection Criteria

We have searched for articles using the above search strategies. Relevant studies, which are in English and have been peer-reviewed (i.e., journal articles and conference papers) were selected for inclusion in this study.

1) Inclusion Criteria

We included studies that met the following criteria:

- Studies that express the theoretical and evidence-based concept of mathematical learning for visually impaired students.
- Studies that contain AT-based solutions to help visually impaired or blind people learn mathematics.
- All related studies that define search terms, answer the research questions and are in the English language were considered for this article.

2) Exclusion Criteria

We have excluded the studies that did not link with learning mathematics, AT and visually impaired or blind people. Studies that were out of scope did not answer the research questions, did not define the search terms or were not written in English were also excluded. Research articles without original data were also excluded.

3) Data Extraction and Assessment of Study Quality

According to our inclusion criteria, all relevant data that fulfilled the requirements and were within the scope were considered for this study. During data extraction and assessment of study quality, we have considered the following points:

- Proper matching of inclusion and exclusion criteria with the selected studies.
- Studies included their findings to help answer the main research questions.
- Literature search has covered all the relevant studies.
- Duplicate articles and articles without empirical evidence.
- The quality of included studies has been assessed properly by authors and reviewers.
- Articles that did not meet the aims and objectives of this study.
- Articles for which the full text was not available.

4) cross-reference the citations

Cross-referencing is a powerful tool that can be used to quickly find related material and enhance our quality of work. We have performed a cross-reference search of the citations to ensure that all relevant studies are included in the review. It helped us in the identification of some additional studies that were not captured in the initial database search and increased the completeness of the review. We repeated this process until a saturation point was reached at which no new relevant information emerged from the literature search, indicating that the search had been comprehensive.

**Fig. 1:**
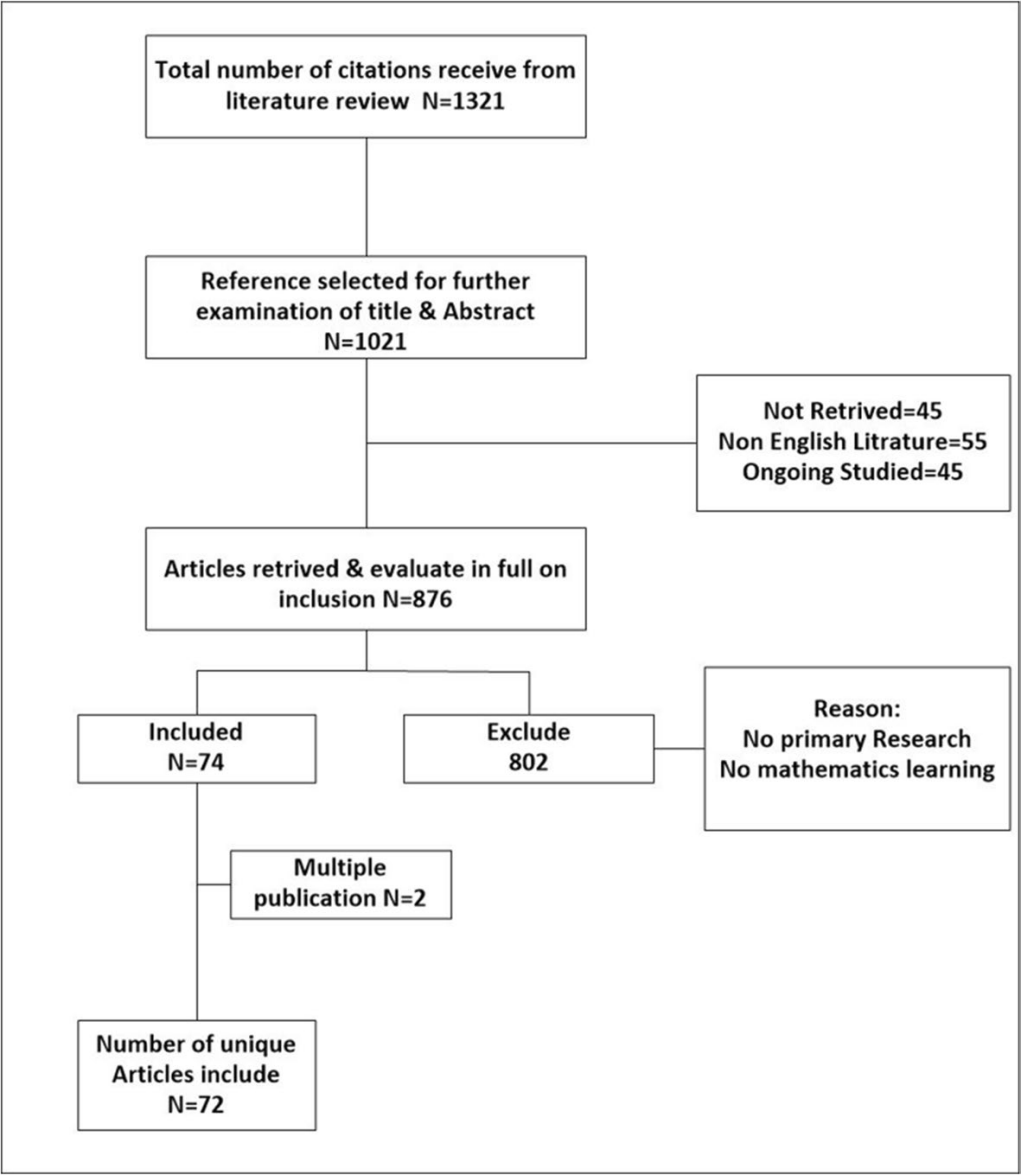
Research methodology

Using the above criteria, we have identified 72 articles published between January 1990 and May 2023. Figure 1 shows the structure of our research methodology and the total number of articles included in this study.

### Classification of the research articles

This section helps to understand the classification of the research articles that are part of the current study.

- *Theoretical literature review*: Studies that help to define, explore, understand and conclude the topic under consideration. It included psychological, physiological, pedagogical and other relevant theories. Studies part of this section do not describe general and targeted solutions.

The papers in this category are considered in Section 3.

b) *Papers which describe practical solutions addressing general issues, with user studies:* These studies address more general issues (e.g., use of speech, non-speech audio or Braille potentially across a range of applications) and have experimental evaluation through participants' involvement. We have summarised them by listing their objectives, participants involvement during experiments and listed their key findings.

The papers in this category are considered in Section 4.1.

c) *Papers which describe practical solutions designed to support specific tasks, without user studies:* These papers all describe solutions aimed at specific problems within mathematics (e.g., learning algebra, exploring graphs, etc.), but none of them include user studies. We have summarised their major features, the mathematical operations they address, and the feedback and input/output mechanisms they employ. This data is presented in the form of a table.

The papers in this category are considered in Section 4.2.

d) *Commercially available applications:* This category covers applications which are available commercially or for free download which have not been described in published research papers, and therefore are not considered under any of the above categories. They include smartphone applications which are available from charitable and educational organisations or through the Google play store or the Apple AStore.

The papers in this category are considered in Section 5.1.

e) Papers which describe practical solutions designed to run on smartphones.
  Only articles describing solutions designed to run on smartphones are included in this category. Most are designed to support specific tasks rather than general solutions. Most of these applications have been evaluated through user studies.

The papers in this category are considered in Section 5.2.

## Background and literature review

In this section, we look at theoretical studies that consider psychological, physiological, pedagogical and other relevant theories, but which do not describe practical solutions, and do not present the results of user studies aimed at assessing how previous research might be applied in the design of solutions. Visually-impaired people face a number of problems during learning mathematics. Several researchers have developed AT-based solutions which may be useful for visually-impaired people in learning mathematics. Mejia et al. surveyed currently available tools that are useful for accessing mathematical content [21]. Armano et al. [22] conducted a study to examine the issues faced by visually impaired students when accessing mathematical content in University Teaching Materials (UTM). They described instruments and software-based learning methods for visually impaired students such as LeanMath, which makes it easier for visually impaired students to use a graphical interface; MathType, which makes graphs more accessible in Word documents with the help of speech synthesis and hotkeys; MathPlayer, which makes formulas more accessible by using MathType; LaTex and BlindMath, which makes TeX easier for visually impaired people to use; LAMBDA, which is used to write math formulas. These solutions empower visually impaired students to write the text along with mathematical content.

Visually impaired students can face trouble in managing structural elements of mathematic formulas.

The Universal Mathematics Accessibility (UMA) [23] system was developed in association with multiple institutes. The main purpose of this system is to make mathematics accessible all over the world. UMA has a translator that converts documents into formats that can easily be accessed by visually impaired people. It provides support for the inter-conversion of different math representations i.e., visual markups and Braille. Visually impaired people can also uniformly access and navigate mathematical expressions independently.

Due to the diverse nature of mathematical notations, it is hard to share documents internationally. LAMBDA [24] was a European project aimed at assisting visually impaired students in accessing mathematics. LAMBDA uses 8 dot Braille code, whereas many countries (e.g., Germany, Italy and France) generally use 6 dot notation. This project had two major components, the first was the LAMBDA code and the second was a mathematical editor. The LAMBDA system offers functional integration of the mathematical code, an editor for writing, and the proper utilization of texts for better understanding [25].

MathPlayer [26] is a free plug-in for Microsoft’s Internet Explorer which offers MathML [27] for visually impaired people. It can also be used with other browsers, e.g., Firefox. It dynamically displays the expression, supports multiple font sizes, provides screen magnification and supports speech generation. It also integrates with different screen readers, e.g., JAWS and Window-Eyes and NVDA.

REMathEx [28] enables visually impaired and blind students to access complex mathematical expressions. The proposed system uses a Braille display and speech synthesis outputs together to offer all necessary information relevant to the mathematical expressions. Users can navigate within the expression easily, and can also modify the expression using REMathEx.

## General and targeted solutions

In this section, we first look at studies which explore the development of mathematical skills among visually impaired and/or blind students through user studies. We then look at tools which are designed to perform specific tasks (e.g., tools for presenting graphs, learning algebra, etc.) concerning mathematics learning among visually impaired and/or blind people. The papers in the latter group do not include user studies.

### Papers which describe practical solutions addressing general issues, with user studies

In this section, we look at studies concerning mathematics learning among visually impaired and/or blind people which have clearly stated research objectives and which were evaluated in practical studies with members of target groups. Table 2 summarises these studies, listing their objectives, participants involvement during experiments and key findings.

**Table 2:** Summaries of previous studies relating to the development of mathematical skills in visually-impaired people

Article/Year /Authors	Objectives of the Study	Participants	Main Findings
[29] 2022 Maćkowski et al	They evaluated eleven detailed assessment categories used in the process of learning mathematics by visually impaired students	Thirty visually impaired students of a high school course take part in this study	They proposed a system with four main elements: elementary division of exercise, checking the results at every stage, performing user interaction, and providing contextual support with the help of hints. The alternative teaching method showed significant improvement in four materials out of eleven: adjusting problems in learning, the appearance of the material, approval (group or individual) and alternative representation of math material. The visually impaired students also reported increased motivation to learn mathematics
[30] 1997 Stevens et al	They used computers to produce multimodal renderings of mathematical information	Speech and nonspeech audio feedback was provided to four blind participants. Six fully sighted participants were used in the evaluation of the task. Twelve fully sighted participants were also used in the Algebra Earcons evaluation	The proposed “Mathtalk” offers multimodal mathematical information accessing mechanism which conveys algebraic structure in synthetically spoken expressions based upon algebra earcons
[31] 2022 Pires et al	They analysed the current practices in mathematics teaching and designed a novel system that supports learning mathematics	Sixteen children were involved in designing and developing tangibles and auditory stimuli	They conducted participatory design sessions with visually impaired students and their teachers. They introduced a novel system to enhance the learning of the students. Sixteen participants took part in this study. They worked on creating tangibles and auditory stimuli to represent numbers, as well as exploring activities to utilize with a tangible user interface. The results demonstrated that their mathematical abilities improved
[32] 2014 Ferreira et al	Provides a game-based learning environment that motivates visually impaired students to learn mathematics	Experiments were conducted on six visually impaired students attending two different schools who were divided into three groups. Group1: 1 Blind and 1 Low vision, Group2: 1 sighted with no feedback, Group3: 2 visually Impaired and 1 sighted	2D spatialized auditory feedback was used here so it can be played without seeing the graphics. The authors used the methodologies of Virvou et al. and Ketamo [33, 34] to test the game which consists of evaluation quizzes before and after the study. The results demonstrated that this game has a positive impact on learning mathematics
[35] 2018 Maćkowski et al	Proposed a technique used for interactive decomposition of math problems, and provides an alternate representation of formulas that were accessible for visually impaired students	In this study, around 1000 sighted and 40 visually impaired students from six different departments of the university used this platform	They introduced a set of rules for describing mathematical formulas after consultation with mathematicians and teachers of visually-impaired people. A web-based application was developed, using PHP and JavaScript. Study results demonstrated that this platform provided a better understanding of mathematical formulas as compared to previously used Braille’a notation based upon translations of conventional mathematical notation
[36] 2011 Crollen et al	This article aimed to explore finger numeral representation to check ‘general cognitive abilities’, ‘use of finger counting and finger monitoring strategies’ and ‘habits of finger counting and finger monitoring’	Three test sets were examined to check general cognitive abilities, fingeruse strategies and habits of finger counting and monitoring by visually impaired people. A total of 14 congenitally blind, and 14 matched sighted children with an age range between 7–13 years participated in this study	Visually impaired children used their fingers less spontaneously as a way to count and show the different quantities. The results demonstrated that in the absence of vision they introduced a typical finger numeral representation, and suggested that the use of canonical finger-counting depends upon the visual recognition of particular hand shapes
[37] 2012 Giesen et al	The main objective was to determine whether a more supportive academic environment can enhance the development of mathematical skills in students with visual impairments	Data from the Special Education Elementary Longitudinal Study (SEELS) was analyzed and examined to measure mathematical achievement. This is a six-year study based on a sample of approximately 13,000 special education students ages 6 to 12. The authors limited their study to 292 visually impaired students	Results showed that good academic support enhanced the learning process, and the level of academic support was directly associated with the mathematics achievement of visually impaired students. But where a cognitive disability was present, both academic support and specialized support were required to improve achievement in mathematics
[38] 2012 Klingenberg et al	The main purpose was to investigate the mechanism by which two students read Braille and try to complete a geometric task. Also, analyze how they shaped the mental representations of the same objects	Experiments were conducted on two visually impaired students aged 10 and 11 years, both of whom had good skills in mathematics and reading Braille. A case study approach was used and data was collected	The two students performed different body movements and postures associated with the shapes of different objects. They rotated with objects. Normally they rotated and experimented with three-dimensional objects. During this process, continually geometric properties were measured i.e., length, area, and volume to “see” the shapes of objects
[39] 2017 Leo et al	An important objective was to explore the benefits of using tactile displays to help visually impaired students develop spatial skills	Four training sessions were conducted with 16 visually impaired participants aged 6–22 years	The results demonstrated that visually impaired students were able to enhance their spatial skills considerably by using programmable tactile graphics. It was observed that learning was enhanced by using the experimental system. It was also noticed that there was no significant performance difference between blind and low-vision students
[40] 2016 Bouck et al	The main aim was to examine the mechanism used to access algebra with the help of a digital textbook in 5 high school visually impaired students	They mainly used qualitative research methodologies on 5 visually impaired students in an algebra class aged between 16–22 years	Visually impaired students showed a dependency on traditional methods of instruction i.e., large print and Braille. Their instructor motivated them to use the digital textbook. After the instructor's inspiration, students were able to use the digital textbook for learning algebra and were able to access the content as easily as when using traditional methods
[41] 2015 Huang et al	Proposed a novel e-learning technique to help visually impaired students learn mathematics skills	They conducted interviews with 12 visually impaired students and their teachers. Also, arranged the assessment session on different four days and then introduced DAISY E-textbook to the students	With the help of a newly designed mathematics curriculum along with the integration of the Digital Accessible Information System (DAISY), the student accuracy rate improved. The authors also mentioned that student proficiency in learning mathematics was significantly enhanced
[42] 2014 Mukherjee et al	The authors proposed a new technique that provided tactile mapping of geometry diagrams on low-cost Braille printers	They provided one-month initial training to 16 visually impaired users to enhance their skills in perceiving Braille characters. Four sighted teachers were also part of this study	Visually-impaired students can usually recognize Braille diagrams but find it difficult to understand them in a short time. By using the experimental system, visually impaired students were able to convert the geometry word problem into a Braille printout. Findings demonstrated that this was a useful tool for teaching/learning geometry diagrams

### Papers which describe practical solutions designed to support specific tasks, without user studies

In this section, we have considered only those research-based studies that have important activities, mathematical operations to solve, feedback mechanisms and input/output associated with them. We considered tools, techniques, algorithms, systems and projects for this purpose i.e., Electrostatic haptic touchscreen system, The Haptic Deictic System, Speaking Math, AfL system, MathSpeak, ASTER, Mathtalk, CONGRATS, Mathgrasp, MATHS and MAVIS.

An electrostatic haptic touchscreen system [43] was used to present dynamic, graph-based data to visually-impaired users and device experts. A total of 12 visually impaired users aged between 9–50 years participated in this study. The results demonstrated that users were able to find the haptic points accurately and developed effective patterns of interaction while using the touch screens. Some haptic components were positioned at the corners of the screen, and these were easily explored by users.

The Haptic Deictic System (HDS) [44] enables visually impaired and blind people to understand the instructor's gestures in the classroom with the help of a haptic glove and cameras. The haptic glove fingertip also facilitates the replacement of visual information during reading. The authors conducted an exploratory study with four visually impaired and two sighted students who have teaching experience and acted as their guides. With the help of the experimental system, an instructor could be confident that all students were following the lecture content, able to tell when students were confused and could verbalize the information represented in the graphs. All of the visually impaired students reported that they could easily understand mathematical concepts when using the system and that the system was not annoying for them.

A computer-based VISO (Voice Input, Speech Output) calculator [45] assists visually impaired students in solving computational problems in mathematics. A single-subject design was used for the training, assessment, and intervention of three visually impaired students of ages 18–19 years. The findings suggest that the time and average number of attempts to solve problems significantly decreased when using the VISO calculator. The visually impaired students reported that problem-solving was easier when using this calculator as compared to other typical means of calculation. Lastly, participants reported that they would like to have an accessible graphing calculator.

Adaptive Content with Evidence-based Diagnosis (ACED) [46] allows visually impaired people to explore the usability of the AfL (Assessment for Learning) system to access algebra content using audio-tactile graphics. It was used to support four visually impaired students, aged 17–29 years, in learning algebra. Participants were interviewed before and after the study. The results showed that users felt comfortable with the audio and interactive tactile graphics. All participants gave positive feedback on the accessible features of the AfL system.

Isaacson et al., [47] designed an algorithm for enhancing the synthetic speech used in the MathSpeak application. They performed efficiency testing on a developed algorithm by considering some parameters i.e., quality of synthetic speech, reception accuracy and MathSpeak processing capacity. Six visually impaired and twenty-one sighted students took part in this study. The results demonstrated that the use of the experimental algorithm improved the performance of MathSpeak, and the capabilities of participants to perform mathematical activities to fulfil their needs were enhanced.

ASTER [48] provides visually impaired people with access to technical documents using auditory representations of the content. Various rendering rules are written in AFL (Audio Formatting Language) to produce different views of the information. OCR-based document recognition was used to browse the internal structure of a document which provides appropriate information to the users. ASTER was implemented using Lisp-CLOS along with an Emacs front-end to present several types of content to visually impaired users.

The Mathtalk system [49, 50] enables visually impaired people to read algebraic expressions and provides speech and non-speech-based ways to interact with the user interface. This was developed in three phases: presentation of the information based upon speech; addition of browsing to identify the best control features in the reading process and inclusion of an audio glance to facilitate rapid audio access to information. A variety of interactive features are introduced to facilitate visually impaired people i.e., auditory mode, direct manipulation style interaction and information flow control. With the help of these features, Mathtalk transforms passive listeners into active readers.

CONGRATS [51], proposed by T.V Raman, is designed to provide a wide range of aid to visually impaired people in plotting and scanning curves. Users can build a computer display either by selecting the options or by specifying the equation of the curves. Once the curve is drawn on-screen, users can access it using audio scans. With the help of sound, a large amount of information can easily be accessed by visually impaired people.

Mathgrasp [52] is a nonvisual application that combines spatial sounds and gestures to allow visually impaired students to manipulate algebra notation. Algebraic expressions are divided into parts, and visually impaired students can access them using synthetic speech. Gestures are very important during conversation with others, so gestural interaction was used to investigate the effect of body movement on gestural interfaces. Users have variations in their gestures, so algorithms were developed to recognize them. The authors introduced gesture-based interfaces to manipulate the algebraic equations and displayed them on a (vertical) auditory spatial display.

The MATHS [53] project was an initiative of the Commission of the European Communities TIDE (Technology Initiative for Disabled and Elderly People) programme. It aimed to provide better access to reading and manipulation of mathematics equations for blind and visually impaired students. It could be used for educational, occupational and personal purposes. It was quite complex to achieve the functionalities of each aspect, so a four-layered approach was used: distributed layer, cognitive layer, perceptual layer and mechanical layer. The authors enhanced the mechanism through which the visual representation was assessed using spatial audio. Second and third-level students could easily read and practice mathematics on MATHS workstations according to their educational preferences, making it useful for a wide range of potential users.

MAVIS [54] was developed using logic programming. The main goal was to facilitate visually impaired students in reading and writing complex mathematical notations. The best educational mechanism for visually impaired students requires two-way communication, visual or printed output for sighted instructors and quality tactile materials or audio feedback for visually impaired students. This is a comprehensive approach which facilitates users and provides more convenient access to the information. Table 3 provides a detailed comparison of the tools, techniques, algorithms, systems, and projects discussed in the proceeding text.

**Table 3:** Comparison of mathematical tools, techniques, algorithms, systems, and projects that have solved some mathematical operations and used specific feedback

Systems, Tools and Projects	Important Activities	Solving Mathematical Operations	Feedback Used	Input	Output
Electrostatic haptic touchscreen system [43]	1. User-centered approach 2. Tanvas electrostatic haptic touchscreen 3. Haptic localization task 4. Usability study	Visualizing graph-based information	1. Haptic 2. Auditory	1. Single finger 2. Static UI elements 3. Graph objects	1. Haptic representation 2. Textures shape representations
The Haptic Deictic System (HDS) [44]	1. Conjunction of speech with fingertip reading 0.2. Use of haptic gloves to replace the visual information 3. Use of phrase charade game to check the capacity of active engagement (skill training game)	Making instructor gestures accessible in the math class	1. Speech 2. Haptic	1. Teacher gestures 2. student’s fingertip reading 3. camera video	1. Vibrating actuator array to guide the user 2. Awareness of both teacher and student pointing info with speech
Speaking Math: Voice Input Speech Output (VISO) [45]	1. Computer-based VISO calculator 2. Speech recognition function based upon MathSpeak 3. Support scientific calculator operations (e.g., roots, log, sin)	VISO calculator to solve computational problems in mathematics	1. Speech	1. voice input 2. Mouse 3. Keyboard short keys	2. Speech output of the solutions
Adaptive Content with Evidence-based Diagnosis (ACED) based upon AfL system [46]	1. Regular mode 2. Low vision 3. Blind mode 4. Identification of the mode 5. Use of adaptive sequencing for Geometry	Access algebra content using audio-tactile graphics	1. Speech 2. Synthetic Speech	1. Text and Pictures 2. keyboard 3. Touch Graphics	1. Magnified data 2. Prerecorded speech
MathSpeak syntactic speech rendering [47]	1. Data for algorithm i.e., teacher recordings and analysis of pauses 2. TTS rendering of expression information 3. Audio instructions of the math rules	Designing an algorithm to increase the quality of syntactic speech rendering	1. Synthetic Speech 2. Auditory	1. Math expression recordings of teachers 2. Text-based math	1. Spoken mathematics 2. Audio rendering of data
ASTER (Audio System for Technical Reading) [48]	1. Recognizing high-level document structure 2. AFL: Audio Formatting Language 3. Rendering rules and styles 4. Browsing audio documents	Quasi-Prefix notation was used to hold the content and structure of the math formulas	1. Synthetic Speech 2. Non-speech	1. TeX, LaTeX and AMS-TeX	1. Audio renderings of electronic documents
Mathtalk (Promotes active reading of content and structure of algebraic formulas) [49, 50]	1. Speaking Algebra Notation (Present the information in speech) 2. Controlling the Information Flow (Add browsing) 3. The Audio glance (*algebra earcons*)	Reading of algebra notation	1. Speech 2. Non-speech	1. Own plain text format	1. Audio access to the information
CONGRATS (Convert Graphics into Sound) [51]	1. Develop a computer display by using audio scans 2. Develop a functional representation of the curve display 3. Interactively plotting and scanning curves	Learn Geometry using sound cues	1. Sound cues 2. Synthetic Speech 3. Speech	1. Geometry Objects	1. Auditory information of curve displayed
Mathgrasp (Algebra manipulation tool which used spatial sound and manual gestures) [52]	1. Extension of Mathtalk by using spatial and structural information 2. Gesture-based model to handle the algebra notations 3. Algorithm to solve the problem of segmentation and the use of unnatural gestures	Algebra expressions manipulation	1. Synthetic speech 2. Non-speech 3. Haptic	1. Gestures and spatial sound	1. By using spatial sound and manual gestures provided a direct manipulation interface for algebra
MATHS (Provides usable access to reading and manipulating mathematical expressions) [53]	1. Recognition 2. Syntactic discrimination 3. Interpretation 4. Glancing and browsing 5. Manipulation and Editing	Mathematical problems of reading and writing. Also, manipulating algebraic expressions	1. Speech 2. Non-speech	1. Reading through visual, tactile and auditory media	1. Auditory information of a wide range of algebraic expressions
MAVIS (Provides easy access to complex math equations) [54]	1. Translate LaTeX to Nemeth code 2. Nemeth Code to LaTeX translation 3. Developed a screen reader for mathematical Information 4. developed the tool for reading and creating the mathematical equations	Reading and writing complex math equations	1. Speech 2. tones	1. Latex markup language and Nemeth Code	1. Translate Latex to Nemeth Code, Latex/Nemeth Code to audio

## Commercial and research-based smartphone solution

In this section, we have described commercial and research-based applications. Listed the features and categorization of available solutions. Additionally, discussed a conceptual model of learning mathematics.

### Commercially available applications

The papers discussed above describe research studies, some of which have yielded tools and systems which are available for free download or through charitable and educational organisations. In addition, there are several computer-based, iOS and Android-based commercial applications which are designed to aid visually impaired students in learning mathematics. Most apps use a voice support function which allows the users to have action awareness within the app. These apps are available for all age groups and are generally easy for visually impaired and blind people to use. Table 4 lists various iOS and android based applications along with features, findings, challenges and issues.

**Table 4:** Describes the features, platform, comments, challenges and issues of the applications

Name of Application	Features	Platform	Comments/ Challenges/ Issues	Developers
Math Melodies^a^	Math Melodies facilitates visually impaired students to understand mathematics easily. With the help of the Voice Support function, it provides audio feedback to users. This application has multiple modules i.e., video-game and math workbook. Math Melodies enables the exploration of audio-visual elements on a touchscreen and provides multi-modal feedback that entertains and engages both visually impaired and sighted children simultaneously. It also has audio icons for all elements of the user interfaces	Android and IOS	The interaction mechanism of Math Melodies has been changed with the help of the Voice Support function and it has been fully translated into English. In this application, some information presented in visual form may cause problems for visually-impaired people	Retina Italia Onlus
Math Robot™^a^	Math Robot was designed especially for blind and visually impaired students but sighted students can also use it. It provides entertaining exercises for simple math problems. This application can be accessed by VoiceOver and self-voicing functions. It also has some important functions i.e., low vision mode, braille support and retina graphics	IOS	Math Robot aworks well, particularly in low vision mode where users can easily choose a problem. The developer has fixed the screen appearance issue which affected some devices and introduced a feedback button in the app. Additionally, it’s better to introduce the word problems in the coming version	American Printing House for the Blind (APH)
TouchMath Counting^a^	TouchMath Counting aintroduces a multisensory system that uses the actual numbers from 0–9 and has corresponding TouchPoints to the digit's value. With the help of TouchPoints, students can learn the real numbers 0–9, number values, counting patterns, and quantity association. It provides the facility to make critical mathematics concepts appealing and accessible for the students	Android and IOS	They have fixed some bugs and made some internal improvements such as making the numeric keypad buttons work properly, and the voice of DIGIT the robot has been re-recorded with higher quality. Some improvements are required i.e., easy navigational mechanism, independent learning, introducing math challenges and counting objects	Innovative Learning Concepts Inc
UAbacus^a^	UAbacus allows users to perform addition and subtraction using the "logic method". Users who want to learn abacus computation can practice and enhance their learning skills by using this app. They can get help step by step and verify their answer here. Users can also learn the computing mechanism of the Cranmer abacus	IOS	Instruction in abacus causes some confusion for students but this application provides a clear and concise overview to the new learner with the help of speaking bead numbers and appropriate sound effects. Additionally, some new modules can be introduced which will cover other areas of math. It has a limited selection of accessible features	University of Arizona
Slapstack Math^a^	Slapstack Math uses math flashcards instead of playing cards and is accessible to all users. Users have to choose one of the following game variations: addition, subtraction, multiplication, division, addition and subtraction, multiplication and division or all four operations. Some numbers have associated actions, requiring players to perform a lot of calculations, physical movement and mental association. The app is fully self-voicing and compatible with VoiceOver	IOS	Slapstack Math offers a pleasurable environment for basic mathematics learning. Users can change the speed of play in several situations. It also provides high colour contrast and large print numbers	American Printing House for the Blind (APH)
Draw2Measure Protractor^a^	Draw2Measure Protractor is mainly designed for visually impaired students,allowing users to measure angles easily. Students can place an angle onto the screen of the device and trace the sides of the angle by touching the screen. The awill record the tracing and calculate the angle. If any object does not fit on the screen the student can find the angle by rotating the device. This application has better accessibility which allows students to measure angles by tracing the sides instead of reading the measurements	IOS	Draw2Measure is useful for blind, low vision and sighted students. It measures the angle both in degrees and radians	American Printing House for the Blind (APH)
Practice2Master Fractions^a^	Practice2Master Fractions is mainly used for learning fraction calculations and provides a large number of exercises and problems to practice. Students can practice fraction subtraction, division, addition, and multiplication. It provides machine-generated calculation problems as well as allows teachers to create problems. Students can save the problems for their future practice. It also provides full compatibility with the iPad's native screen reader VoiceOver	IOS	This acan store reports on exercises attempted and provides user-selectable options. Students can exchange problem lists. It also provides large print numbers and high-contrast colours to assist visually impaired students. In future, they can enhance the practice values of learning skills by carefully expanding the design feature i.e. by introducing a self-explanatory element of the interfaces	American Printing House for the Blind (APH)

### Papers which describe practical solutions designed to run on smartphones

Studies describing smartphone-based applications that have been published in research are part of this section i.e., i-Math, iCETA and AudioMath. The i-Math [55] is an automatic math expression reading system used to facilitate visually impaired students in retrieving mathematics material. It works with a screen reader and provides an auditory output on a computer system. Students can access the information and teachers can prepare their handouts and exercises in audio form. A total of 78 visually impaired students and six teachers participated in an evaluation of the system. The results showed that mathematical material is easily available and accessible for visually impaired students using iMath. The students were able to practice the mathematical task independently and comfortably. The iCETA game [56] provides an interactive learning environment to visually impaired children using haptic and auditory feedback. Visually impaired students can easily understand numbers concepts with the help of various representations i.e., color, Braille, haptic and audio feedback. AudioMath used auditory interfaces to enhance the mathematics skills of blind children. Ten children participated in this study. Results showed that with the help of proposed interfaces mathematical skills were improved [57].

### Features of Commercial and research-based math learning applications

In this section, we listed the features of commercially-available applications discussed in Sect. 5.1, and the smartphone-based applications discussed in Sect. 5.2. We have mentioned ten commercially and research-based available applications, namely Math Robot™, Draw2Measure Protractor, Slapstack Math, Practice2Master Fractions, UAbacus, Math Melodies, TouchMath Counting, i-Math, iCETA Tangible Math and AudioMath and extracted their common features. Table 5 lists the names of the commercial and research-based applications along with their feature.

**Table 5:** Shows the applications names and their features

Application Name	Multiple modes	Magnification	VoiceOver support	Braille support	Interactive interface
Math Robot™	✓	✓	✓	✓	✓
Draw2Measure Protractor	×	×	✓	×	✓
Slapstack Math	✓	✓	✓	×	✓
Practice2Master Fractions	✓	✓	✓	×	✓
UAbacus	✓	×	✓	×	✓
Math Melodies	✓	×	✓	×	✓
TouchMath Counting	×	✓	✓	×	×
i-Math [55]	✓	×	✓	✓	✓
iCETA Tangible Math [56]	×	×	✓	✓	✓
AudioMath [57]	✓	✓	✓	×	✓

**Fig. 2:**
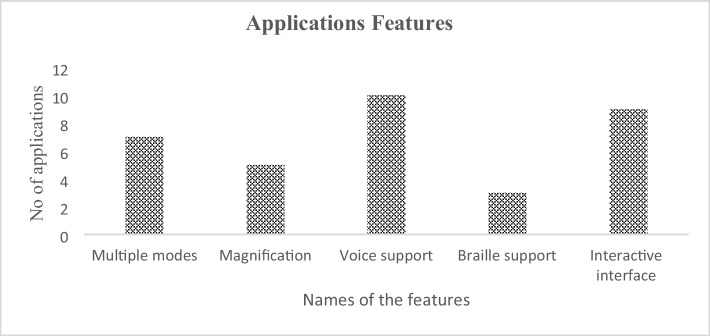
Math learning applications features

Figure 2 shows that seven applications have multiple modes, five applications have magnification, ten applications have voice support function, three applications have braille support and nine applications offer an interactive interface (i.e., easy interaction, clear purpose, high usability, better color contrast and sizes of the UI element).

### Categorization of available solutions

**Fig. 3:**
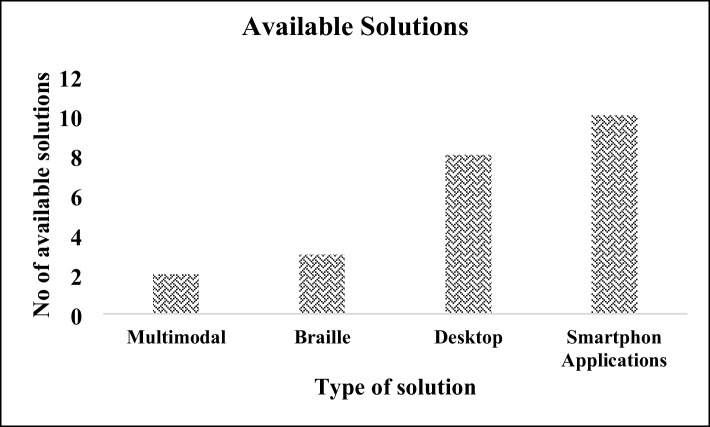
Available solutions in the current study

We have categorized the available solutions described in the papers as Multimodal, Braille, Desktop and Smartphone-based solutions. Multimodal approaches include two solutions, namely REMathEx and LAMBDA. There were three Braille-based solutions, namely 3D printers, tactile graphics and Braille mapping of geometry diagrams. There are eight desktop-based solutions, namely MathPlayer, MathML, LaTeX-access, Speaking Math calculator, Audio Math, AfL system, Mathtalk and Mathematics for all. Ten smartphone-based solutions formed part of the current study, namely Math Robot™, Draw2Measure Protractor, Slapstack Math, Practice2Master Fractions, UAbacus, Math Melodies, TouchMath Counting, i-Math, iCETA Tangible Math and MathSpeak. Figure. 3 showed the information on available solutions in the current study.

### Conceptual Model of learning mathematics

Mathematics plays an important role in education, and every student should have the opportunity to learn mathematics. Learning is a process whereby anyone can enhance his knowledge by transforming his experience. Visually impaired students normally use the auditory and tactile senses in the absence of a visual channel. So, for visually impaired students, the quality of the learning experience depends upon a good teacher and AT-based solutions. Teachers should design the course content, assignments, handout and other activities by using assistive technologies so that learning becomes easier.

**Fig. 4:**
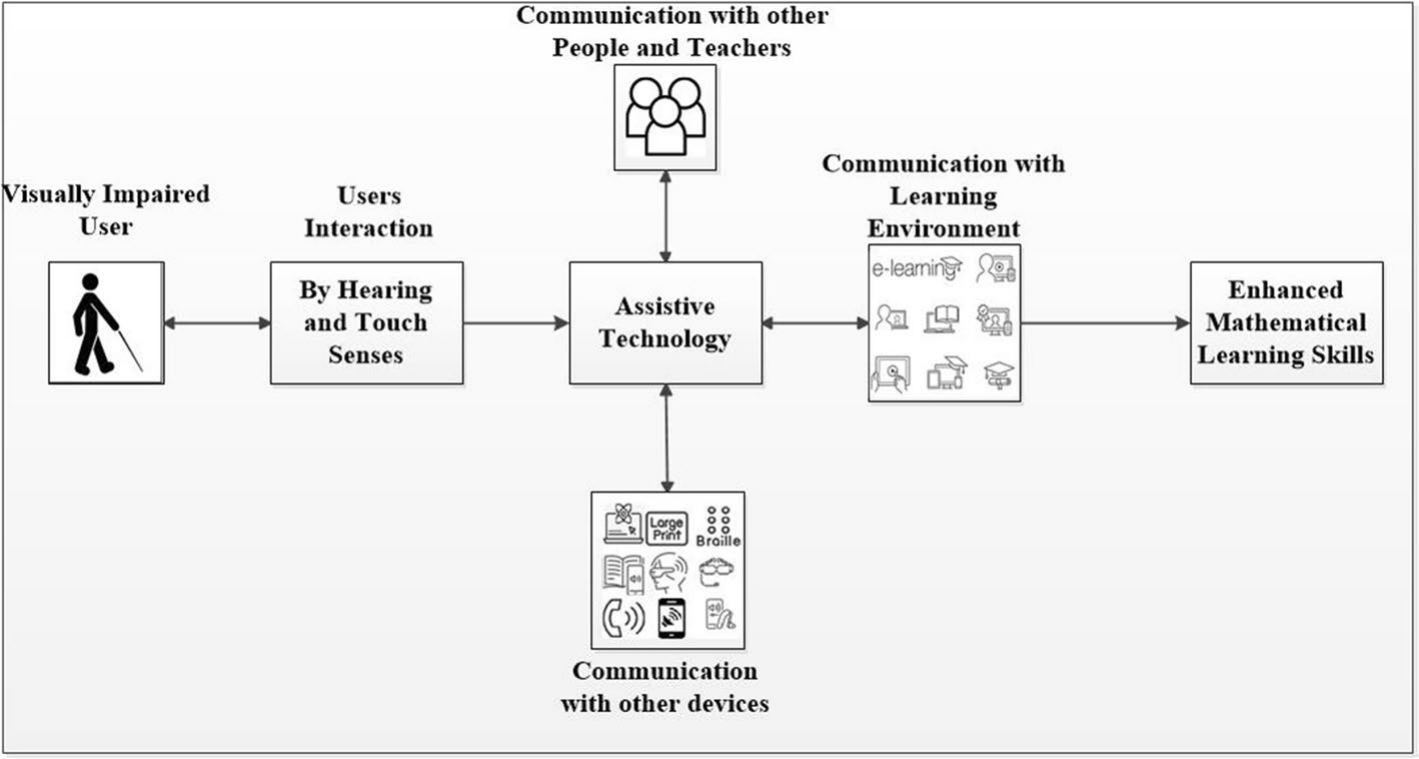
Conceptual model of learning mathematical skills by using AT

Figure 4 provides a conceptual model of learning mathematical skills using AT. They can communicate with other devices, the learning environment, other people and their teachers by using AT. This model shows that AT empowers visually impaired users to significantly enhance their mathematical skills.

## Discussion

Previous research has shown that AT provides the opportunity for visually impaired and blind students to enhance their learning process by using accessible mathematical materials. This discussion section has three parts to elaborate on the impact of technology on the development of mathematical abilities among visually impaired and blind students.

1. The first part provides an overview of teaching mathematics to visually impaired people
2. The second part is about the applications and their features for learning Mathematics in AT.
3. The third part presents a categorization of available solutions.

Firstly, a systematic review of previous studies was conducted. Most studies were evaluated on visually impaired and/or blind participants. The findings showed that accessible math materials(material that is in a format which is optimised for accessibility), academic support, haptic feedback, tactile graphics, digital books and audio-based platforms are very useful to address the challenges of visually impaired people. Earlier research also demonstrates that mathematical material can be made easily understandable for visually impaired students, and by using that material their learning process can be enhanced [58, 59]. It is a challenging task for visually impaired students to access information presented in graphic form. This information can be accessed using AT-based solutions where the focus is shifted towards hearing and touch-based techniques. The comparison of tools, techniques, algorithms, systems and projects provides a detailed overview of the current solutions used for enhancing the mathematical skills of blind and visually impaired people. These solutions offer several functionalities such as: converting graphics into sound, syntactic speech rendering, audio-tactile graphics, designing gestural interfaces and introducing audio-based mathematical reading systems (i.e., technical content and mathematical formulas). We have also listed the feedback mechanism, and the input and output mechanisms used by each system used to handle different mathematical operations i.e., visualizing graph-based information and accessing algebra content using audio-tactile graphics. It is also found that users can access math formulas, algebra expressions, complex math equations and geometry shapes using these solutions. Several forms of feedback were used in the designing of these solutions i.e., synthetic speech, non-speech, haptic, sound cues and tones. It is also noticed, that whenever spatial sound, auditory and hepatic feedback is available along with visual and textual information then visually impaired users can easily perceive the information. (RQ1).

Secondly, we have discussed commercial and research-based applications of learning Mathematics in AT. These applications present the graphical information of mathematics to visually impaired students using Braille notation, tactile displays, interactive interfaces, screen readers, voice synthesizers and zooming programs. The voice support function of the applications is quite useful for visually impaired and blind people. They can access mathematical formulas and solve problems using this function. Research shows that applications with the combination of audio and tactile feedback significantly enhance the process of learning mathematics. We have also explored the features of available applications such as voice support function, audio-visual elements, multi-modal feedback, audio icons, self-voicing functions, low vision mode, braille support, multisensory support and math flashcards. These features are available in current applications to facilitate visually impaired and blind students. We have also provided a comparison of some common features of the applications i.e., interactive interface, multiple modes, magnification, voice and braille support. Comparison results showed that every application has a voice support function and with the help of this function, visually impaired students can access the material easily. Visually impaired and blind students faced several challenges during the use of commercial and researcher-based applications. For example, applications are not designed according to the accessibility features and are unable to cater to the needs of visually impaired and blind students i.e., problems with feedback (lack of screen reader). It is noticed that some applications are not designed in a way that blind and visually impaired students can use them easily i.e., have rich visual information and complex navigation mechanism. Some applications have limited functionalities which can make it challenging for these students who are looking for a solution that provides support to them on various topics of mathematics.(RQ2).

Thirdly, the categorization of the available solutions is described as Multimodal, Braille, Desktop and Smartphone-based solutions. These categorizations clearly showed that smartphone-based solutions are more prominent and there is a lack of multimodal approaches to provide support to visually impaired and blind students in learning mathematics. On the other hand, researchers' intentions towards developing smartphone-based solutions are quite high as compared to Braille and computer-based solutions. Mostly visually impaired people can easily afford mobile devices and have satisfactory experiences with them. Also, several useful and free applications are available from astores. With the help of smartphone-based solutions, users educational abilities could enhance and their learning behaviour can be significantly improved. It was also noted that educational material supported by smartphone-based applications gave a positive impact on the learning outcomes of visually impaired students. Smartphones are also equipped with accessibility functions that provide easiness to visually impaired people in accessing information. (RQ3).

## Conclusion

We have concluded in this article that AT facilitates visually impaired and blind people in learning mathematics and provided a detailed review of some existing tools, techniques, applications and games. Developments in e-learning have proved beneficial for educational enhancement, but also pose challenges for visually impaired students [60–63]. Research has shown that various challenges and issues were overcome using AT-based solutions, i.e., accessing graphical information, lack of independent learning, high color contrast and large print numbers. We have found that unimodal learning systems are less effective than systems that offer multimodal approaches to access math resources. In multimodal techniques, we can combine different approaches i.e., Braille, synthetic speech, tactile visual display, tangible resources and tangible user interfaces. With the help of innovative technologies, various computer-equipped solutions have been developed, such as a GUI calculator and a virtual learning platform for the web environment [64, 65].

Additionally, mobile devices and app-based interventions are very useful, affordable and have a positive impact on the educational system [66]. The smartphone-based solutions also provide multiple representations of information i.e., audio and tactile feedback, zooming, pictures, video, animation, user control, and repetition of tasks, and can serve to create a personalized learning environment [67–69].

Furthermore, the learning environment, technology-based training, student motivation and teacher expertise play an important role in the learning of visually impaired and blind people. It is also recommended to have academic, technological and specialized support for visually impaired and blind people learning mathematics. Visually impaired and blind students prefer applications that allow them to practice mathematical tasks independently and comfortably. We have seen that AT-based solutions can minimize several challenges faced when accessing graphical and visual content in mathematics, and enhance problem-solving skills in visually impaired and blind students.

Moreover, different study designs, data collection and analysis approaches were used in previous research i.e., qualitative research methodologies, UCD, usability studies, exploratory studies, single-subject designs and efficiency testing of algorithm parameters. Previous research studies showed that interviews, questionnaires and participant observation were very useful for the assessment of studies. Pre-study training sessions gradually enhance the skills of visually impaired and blind people and are also helpful for their learning intervention.

The research reviewed here suggests that provided the instructor successfully motivates students regarding the use of digital textbooks, it can be more useful for them to access mathematics material in this way rather than using traditional methods. Finally, earlier studies showed that AT-based solutions have a positive impact on the learning skills of visually impaired and blind people. Assistive and innovative technologies are also very useful for overcoming challenges and issues facing visually impaired and blind people. The development of smartphone-based solutions empowers visually impaired and blind users by providing them with accessible material on mathematics.

Lastly, we have introduced a conceptual model of learning mathematical skills using Assistive Technology (AT). This model shows that AT empowers visually impaired users to significantly enhance their mathematical skills. We have categorized the available solutions described in the papers as Multimodal, Braille, Desktop and Smartphone-based solutions that could be helpful for readers and other researchers. We have discussed the commercially-available and research-based applications. Extract their features i.e., multiple modes, magnification, voice support function, braille support and interactive interface. We have identified the challenges/issues faced by visually impaired and blind students using current solutions and provided this information in the discussion section which is very useful for other readers. We are trying to encourage researchers to get familiar with the unsolved challenges of this topic.

Researchers and adevelopers should keep in mind that every visually impaired and blind student has a different level of knowledge and intellectual abilities, Therefore, newly-designed applications should offer learning modules at various levels, integrated with Artificial Intelligence-based modules that can identify the user's abilities and identify the learning level of the users. In addition, adopting a Universal Design approach may help to meet the needs of all users. Artefacts developed using universal design can be accessed and understood by all kinds of users regardless of their abilities, knowledge or disabilities. Universal design can provide an accessible, usable, suitable and pleasurable environment to everyone.

There are also some issues and limitations in current applications. Due to these limitations visually impaired and blind students feel difficulty in accessing structural information and mathematical formulas. To address these problems, developers should introduce an ‘ask for help’ module which offers additional clues about the specific task and assists visually impaired students when they cannot remember the next steps. Additionally, desktop-based solutions have some drawbacks such as limited portability, lack of touch screens, increased space and cabling requirements, and access to mains power. Smartphone-based solutions offer advantages for visually impaired and blind people because they are more convenient, less expensive and easily available for everyone.

## Future directions and recommendations

We have provided future directions and recommendations for the development of smartphone-based applications that can be useful in helping visually impaired and blind people in learning mathematics.

Earlier research showed that the focus has shifted from vision to hearing in eyes-free environments [70–72]. Recent innovative technologies use hearing, haptic and multimodal combinations of senses to help visually impaired and blind people learn mathematics. Most of them support only the English language, in future, a solution may be developed which has no language dependency. Therefore, people whose first language is not English may have a chance to develop their mathematical skills easily.

In future, researchers can investigate how math teachers effectively teach advanced-level mathematics skills to visually impaired and blind students using AT. For this purpose, haptic feedback is very useful because, in the absence of vision, it can be used with visual input to construct mathematical learning systems. Lastly, in the field of mathematics, there is a need for more adaptive systems. Developers should introduce adaptive systems and applications which automatically change their configuration and behaviour based on the interaction of the user.
